# *Staphylococcus ratti* sp. nov. Isolated from a Lab Rat

**DOI:** 10.3390/pathogens11010051

**Published:** 2022-01-01

**Authors:** Vojtěch Kovařovic, Ivo Sedláček, Petr Petráš, Stanislava Králová, Ivana Mašlaňová, Pavel Švec, Meina Neumann-Schaal, Tibor Botka, Tereza Gelbíčová, Eva Staňková, Jiří Doškař, Roman Pantůček

**Affiliations:** 1 Department of Experimental Biology, Division of Genetics and Molecular Biology, Faculty of Science, Masaryk University, Kotlářská 2, 611 37 Brno, Czech Republic; 2 Department of Experimental Biology, Czech Collection of Microorganisms, Faculty of Science, Masaryk University, Kamenice 5, 625 00 Brno, Czech Republic; 3 Reference Laboratory for Staphylococci, National Institute of Public Health, Šrobárova 48, 100 42 Praha, Czech Republic; 4 Leibniz Institute DSMZ-German Collection of Microorganisms and Cell Cultures, Inhoffenstraße 7 B, 38124 Braunschweig, Germany; 5 Department of Public Health, Faculty of Medicine, Masaryk University, Kamenice 5, 625 00 Brno, Czech Republic

**Keywords:** laboratory rat, *Staphylococcus*, Hyicus-Intermedius species group, taxonomy, whole genome sequencing, variable genetic element, genomic island

## Abstract

Staphylococci from the *Staphylococcus intermedius*-*Staphylococcus hyicus* species group include numerous animal pathogens and are an important reservoir of virulence and antimicrobial resistance determinants. Due to their pathogenic potential, they are possible causative agents of zoonoses in humans; therefore, it is important to address the properties of these strains. Here we used a polyphasic taxonomic approach to characterize the coagulase-negative staphylococcal strain NRL/St 03/464^T^, isolated from the nostrils of a healthy laboratory rat during a microbiological screening of laboratory animals. The 16S rRNA sequence, MALDI-TOF mass spectrometry and positive urea hydrolysis and beta-glucuronidase tests clearly distinguished it from closely related *Staphylococcus* spp. All analyses have consistently shown that the closest relative is *Staphylococcus chromogenes*; however, values of digital DNA-DNA hybridization <35.3% and an average nucleotide identity <81.4% confirmed that the analyzed strain is a distinct *Staphylococcus* species. Whole-genome sequencing and expert annotation of the genome revealed the presence of novel variable genetic elements, including two plasmids named pSR9025A and pSR9025B, prophages, genomic islands and a composite transposon that may confer selective advantages to other bacteria and enhance their survival. Based on phenotypic, phylogenetic and genomic data obtained in this study, the strain NRL/St 03/464^T^ (= CCM 9025^T^ = LMG 31873^T^ = DSM 111348^T^) represents a novel species with the suggested name *Staphylococcus ratti* sp. nov.

## 1. Introduction

Staphylococci are opportunistic pathogens widespread in nature; they are mainly isolated from the skin, skin glands, and mucous membranes of various animals [1], and less often from the blood of diseased animals [2]. The nasal cavity of many mammalian species is inhabited by distinctive staphylococcal species. Studies of *Staphylococcus* spp. distribution in wild rodents have shown the presence of predominantly coagulase-negative staphylococci, including *Staphylococcus xylosus*, *Staphylococcus equorum*, *Staphylococcus succinus*, *Staphylococcus saprophyticus* and *Mammaliicoccus* spp. [3,4]. The species composition of staphylococcal populations in wild and laboratory rats is largely unknown. The predominant species in laboratory rats is *S. xylosus*, followed by *Staphylococcus aureus* and *Staphylococcus cohnii* [5]. Recent studies have shown that rats are reservoir of various *S. aureus* clonal lineages, including methicillin-resistant strains [6]. On the other hand, murine *S. aureus* isolates exhibit host adaptation [7]. Both mice and rats are an animal infection model for studying *S. aureus* pathogenicity [8,9]. In wound infections, rats eliminated bacteria faster and more rapidly organized the inflammatory response than other rodents [10].

Similarly to the main pathogen *S. aureus*, some species in the *Staphylococcus intermedius*-*Staphylococcus hyicus* phylogenetic complex can also catalyze the polymerization of fibrinogen to fibrin through the enzyme coagulase, thereby contributing to the appearance of purulent foci—abscesses [11]. Besides coagulase, they also have other virulence factors, such as staphylococcal β-hemolysin (sphingomyelinase), a typical product of the species *Staphylococcus pseudintermedius* [12]. Staphylococcal species belonging to this complex are opportunistic pathogens of various animal species. *S. pseudintermedius*, *Staphylococcus coagulans*, and *Staphylococcus canis* occur as carriers in dogs and other carnivores [13,14,15,16], and when skin integrity is disrupted, they can cause skin and soft tissue infections or external ear otitis. *Staphylococcus ursi* was isolated from healthy black bears [17]. *Staphylococcus felis* is a common cat commensal and a potential urinary pathogen [18]. However, they can also be zoonotic pathogens, causing skin problems in humans; a typical example is a septic wound after a dog bite or cat scratch [19,20].

*Staphylococcus hyicus* forms a separate phylogenetic clade together with *Staphylococcus agnetis* and *Staphylococcus chromogenes*. *S. hyicus* causes exudative epidermitis in pigs, known as greasy pig disease, due to the expression of exfoliative toxins that selectively digest porcine desmoglein 1 [21]. Zoonotic infections of the bloodstream have been reported to be caused by *S. hyicus*, as has spondylodiscitis in animal keepers [22,23]. *S. agnetis* was primarily isolated from cows with mastitis [24], but recent findings demonstrate that *S. agnetis* may be associated with diseases and mortality in broiler chickens [25,26]. *S. chromogenes* is globally the most common cause of intramammary infections of dairy cows [27] and has also been reported in both healthy and diseased pigs [28], goats [29] and poultry [5]. There are also reports of nasal carriers of *S. chromogenes* among farm workers [30].

The prevalence of human infections caused by species of the *S. intermedius*/*S. hyicus* complex is low; however, due to their phenotypic similarity, the capture of some species may be underestimated in studies that did not use molecular techniques. In this work we provide a polyphasic characterization of an isolate of *Staphylococcus* sp. NRL/St 03/464^T^ that was cultured during a microbiological examination of nasal swabs from healthy rats before a biological experiment at the Unit for Biomedicine and Welfare of Laboratory Animals, National Institute of Public Health (Prague, Czech Republic).

## 2. Results and Discussion

### 2.1. Phylogenetic Analyses

The isolate CCM 9025^T^ (= NRL/St 03/464^T^) was assigned by a partial sequencing of the 16S rRNA gene to the *Staphylococcus* genus and previously defined Hyicus-Intermedius species group [31]; however, its biochemical profile, MALDI-TOF MS pattern and partial RNA polymerase subunit beta (*rpoB*) gene sequence did not allow for its classification into any known staphylococcal species. Therefore, a polyphasic taxonomic study was conducted which was focused on a detailed characterization of a new *Staphylococcus* species.

**Figure 1:**
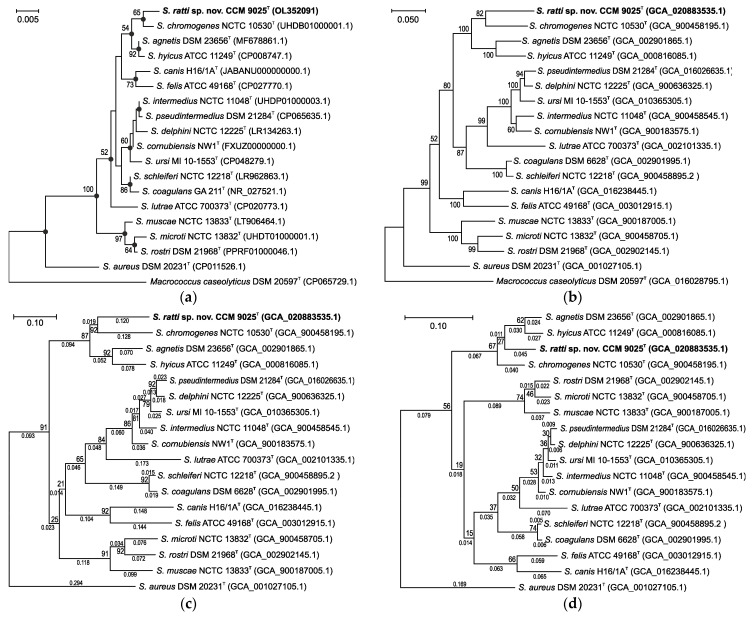
Evolutionary analyses of *S. hyicus*-*S. intermedius* phylogenetic complex including *S. ratti* sp. nov. (**a**) Unrooted phylogenetic tree based on complete 16S rRNA gene sequences extracted from whole-genomic sequencing data (GenBank accession numbers are in parentheses). The evolutionary history was inferred by using the maximum likelihood method and Tamura–Nei model. Filled circles indicate that the corresponding nodes were also obtained in the tree constructed by the neighbor-joining method. The percentage of 500 tree replications above 50% in which the associated taxa clustered together is shown next to the branches. The tree is drawn to scale, with branch lengths measured in the number of substitutions per site. There were a total of 1551 positions in the final dataset. (**b**) Unrooted maximum likelihood tree based on multilocus sequence analysis of concatenated nucleotide sequences from six loci, *rpoB*, *hsp60*, *dnaJ*, *tufA*, *gap* and *sodA*, extracted from whole genome assemblies (accession numbers are in parentheses). There were a total of 3972 positions in the final dataset. Bootstrap probability values (percentages of 500 tree replications) greater than 50% are shown at branch points. The evolutionary distances are given as the number of substitutions per site. (**c**) Nucleotide core gene set phylogenetic tree of *S. ratti* sp. nov. and phylogenetically related species. (**d**) Protein sequence-based phylogenetic tree of the core gene set of *S. ratti* sp. nov. and phylogenetically related species. Trees (c) and (d) were constructed using up-to-date bacterial core gene set (UBCG; concatenated alignment of 92 core genes). The maximum likelihood tree was inferred using RAxML software and set to 100 replicates. Gene support indices are given at branching points (maximal possible value is 92). Bar, 0.1 substitution per position.

The obtained complete 16S rRNA gene sequence of strain CCM 9025^T^ was compared to those of other taxa from the Hyicus-Intermedius species group of the *Staphylococcus* genus. The closest relatives were *S. chromogenes* (99.3% similarity), *S. agnetis* (99.2%) and *S. hyicus* (99.1%); other species were below 98% similarity. The topology of the maximum likelihood phylogenetic tree constructed with 16S rRNA gene sequences was similar to that of the neighbor-joining tree (Figure 1a).

Because the 16S rRNA analysis has limited discriminatory power for the identification of some staphylococcal species, the phylogenetic position of the new isolates was also assessed using the concatenated multilocus sequence data of six routinely used housekeeping genes (Figure 1b). The maximum likelihood phylogenetic trees for the housekeeping genes including the closest relatives had a very similar topology which corresponded to that of the 16S rRNA gene tree, clearly separated the novel isolate CCM 9025^T^ from the established species and which confirmed *S. chromogenes* as the closest relative. The significant phylogenetic distance from the related staphylococcal species at the whole-genome level, with an average nucleotide identity (ANI) of <81.4% and digital DNA-DNA hybridization (dDDH) of <35.3%, were below the species delineation thresholds, which are 95–96% and 70%, respectively [32]. This confirmed that strain CCM 9025^T^ represents a distinct *Staphylococcus* species named *Staphylococcus ratti* sp. nov. It forms a separate branch in the *S. hyicus* phylogenetic clade, as also shown by protein coding core genome analysis using the up-to-date bacterial core gene (UBCG) (Figure 1c,d).

### 2.2. Phenotypic, Genotypic and Chemotaxonomic Characteristics

**Figure 2:**
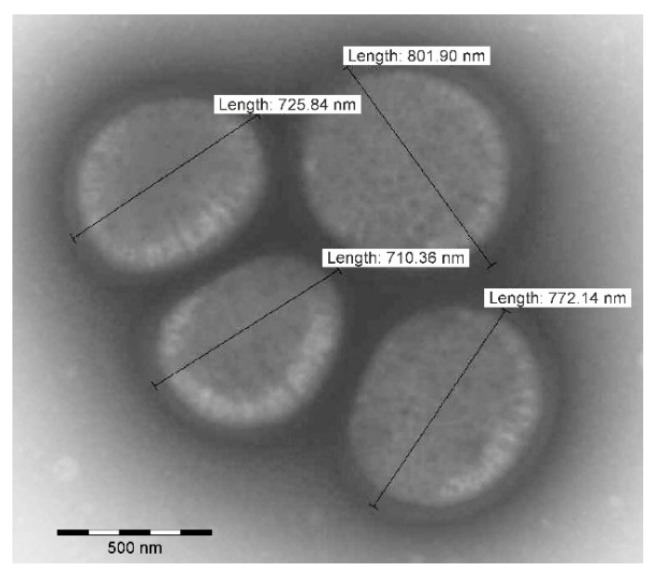
Transmission electron microscopy image of type strain *Staphylococcus ratti* sp. nov. CCM 9025^T^ negatively stained with ammonium molybdate. Bar represents 500 nm (original magnification × 10,000).

Cells of strain CCM 9025^T^ are irregular spherical cocci with diameter 705 ± 55 nm (*n* = 100) (Figure 2). The strain grew well on common media for staphylococci and was mesophilic and moderately halophilic, with the ability to hydrolyze biomacromolecules (gelatin, DNA). Carbohydrates seldom served as the source of carbon. The detailed phenotype data are subsequently mentioned in the species description in the text; here we only specify several notable results. Interestingly, the hyaluronidase test result was positive for CCM 9025^T^, which is a rare feature for coagulase-negative staphylococcal species. During biotyping, a few contradictory results depending on the tested conditions were found. Firstly, the Voges–Proskauer test (acetoin) was negative in a standard tube test, but positive with a commercial VPtest strip containing pyruvic acid instead of glucose. The second inconsistency was related to an enzymatic β-glucuronidase test included in the commercial kits, when β-glucuronidase was positive for CCM 9025^T^ in STAPHYtest 24, but negative in API ZYM, probably due to different substrates being used for enzyme detection.

The tests distinguishing novel species from closely related staphylococci of the *S. hyicus* group are shown in Table 1, and these tests enable the correct identification of CCM 9025^T^ at the species level.

Profiling analysis by matrix-assisted laser desorption/ionization—time-of-flight mass spectrometry (MALDI-TOF MS) is routinely used for bacterial identification. A distinct MALDI-TOF MS pattern is therefore a useful phenotypic feature in describing new species. Strain CCM 9025^T^ generated a consistent MALDI-TOF MS profile containing signals in the mass range of 2–10 kDa, which was not related to any of those *Staphylococcus* species already represented in the commercial database at the time of testing. After the manual inclusion of profiles from 24 analyses of the strain CCM 9025^T^ to the in-house database, the strain was re-analyzed and matched the new pattern with a score of 2.6, whereas the next closest species was *S. schleiferi* with a score of 1.2, which is far below the species identification threshold.

Staphylococci are easily differentiated from other Gram-positive cocci at the genus level by cellular fatty acid analysis, which is also one of the recommended tests at the species level. The cellular fatty acid profile of strain CCM 9025^T^ revealed 4 major fatty acids (FAs) (≥10%), iso-C_15:0_ (35.0%), anteiso-C_15:0_ (24.1%), iso-C_17:0_ (10.8%) and anteiso-C_17:0_ (10.2%), similar to other validly named *Staphylococcus* spp. [33]. Comparison to the closest related *Staphylococcus* spp. showed qualitatively similar profiles of FAs with quantitative differences between the compared type strains (Table 2). Qualitative differences in iso-C_15:0_, iso-C_17:0_ and C_20:0_ clearly distinguish strain CCM 9025^T^ from *S. chromogenes* CCM 3387^T^, and lower amounts of anteiso-C_17:0_ are specific for *S. agnetis* CCM 8869^T^. *S. hyicus* CCM 2368^T^ has the most similar FA profile to CCM 9025^T^, but can be distinguished by lower amounts of branched C_17:0_ FAs and a higher amount of iso-C_15:0_.

The major respiratory quinone in strain CCM 9025^T^ was MK-7 (95.7%). Menaquinones MK-6 and MK-8 were also detected as minor components of the electron transport system. The identification of MK-7 as the major component of the quinone system is in accordance with the genus characteristics, as members of the genus *Staphylococcus* reveal the presence of unsaturated menaquinones, typically with six, seven or eight isoprene units [33,34]. Analysis of the peptidoglycan structure revealed a cross-linkage type A structure of A3α L-Lys-Gly_3-4_ similar to the type A11.2 structure [35], with the molar amino acid ratio 2.0 Ala:3.4 Gly:1.0 Glu:0.8 Lys. The type A11.2 peptidoglycan structure was also identified in the closest related species *S. chromogenes*, *S. agnetis* and *S. hyicus*, as described by Schumann [35]. Unlike CCM 9025^T^ and *S. agnetis*, *S. chromogenes* and *S. hyicus* were found to also contain minor amounts of serine, likely substituting for some glycine in the interpeptide bridge [24,33,36].

**Figure 3:**
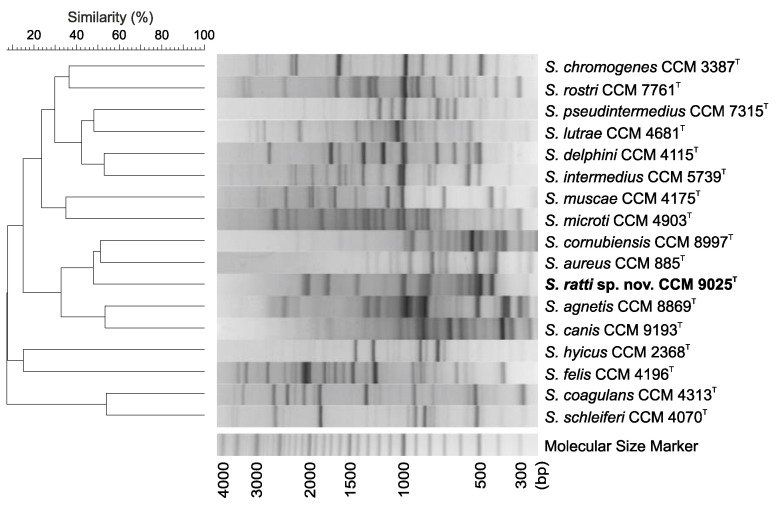
Distinct DNA banding patterns obtained in identification of *Staphylococcus ratti* sp. nov. and the type strains of related species based on repetitive PCR fingerprinting with (GTG)_5_ primer. The dendrogram based on cluster analysis of rep-PCR fingerprints was calculated with Pearson’s correlation coefficients with an unweighted pair group method with arithmetic average (UPGMA) clustering method (*r*, expressed as percentage similarity values).

A DNA fingerprinting technique using repetitive sequence-based PCR (rep-PCR) fingerprinting with the (GTG)_5_ primer, previously shown to be suitable for the simultaneous detection and differentiation of *Staphylococcus* spp., was used to demonstrate the difference of strain CCM 9025^T^ from related taxa. The rep-PCR clearly distinguished the analyzed strain from the type strains representing the phylogenetically close *Staphylococcus* spp. (Figure 3).

### 2.3. Whole Genome Characterization of Staphylococcus ratti sp. nov.

The genome of *S. ratti* sp. nov. type strain CCM 9025^T^ was sequenced using Illumina and Oxford Nanopore platforms. The size of the complete chromosome assembly is 2.3 Mb with a mean coverage of 500-fold. Based on the NCBI automated annotation pipeline, a total of 2198 CDSs were identified in the genome, of which 2150 were protein-encoding genes. A total of 82 genes for RNAs were identified in the genome, including 59 tRNAs, 19 rRNAs including 7 (5S), 6 (16S) and 6 (23S), and 4 ncRNAs. Two plasmid sequences named pSR9025A (3311 bp) and pSR9025B (2455 bp) were assembled and annotated as separate extrachromosomal replicons. Plasmid pSR9025A encodes the gene for the Rep protein, which shares 100% amino acid identity with the Rep protein gene in p908 of *S. agnetis* [37] and a *gsiB* (glucose starvation-inducible protein B) gene homologue which is involved in a stress response. Further short similar regions were identified in the plasmids of many other coagulase-negative staphylococci. The plasmid pSR9025B is a cryptic plasmid similar (75.5% identity and 27% coverage) to the pLNU9 plasmid of *S. chromogenes* [38]. This is an indication of interspecies transfer of these types of variable genetic elements (VGEs).

**Figure 4:**
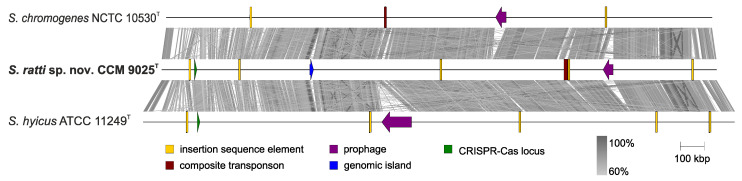
Whole-genome alignment of *Staphylococcus chromogenes* NCTC 10530^T^ (assembly accession no. GCA_900458195.1), *Staphylococcus ratti* sp. nov. CCM 9025^T^ (GCA_020883535.1) and *Staphylococcus hyicus* ATCC 11249^T^ (GCA_000816085.1) chromosomes. Blast map shows nucleotide sequence identity above 60%. The location of variable genetic elements is color coded as in the legend.

Comparative genomic analysis of *S. ratti* with the two type strains *S. chromogenes* NCTC 10530^T^ and *S. hyicus* ATCC 11249^T^ revealed the presence of additional VGEs, including insertion sequence elements, a composite transposon, one prophage, and a genomic island (Figure 4).

Type I restriction-modification (RM) system genes were found downstream of *orfX*, but no evidence of staphylococcal cassette chromosome (SCC) integration was found. Type II RM system genes are localized near a *cap* operon. The clustered, regularly interspaced, short, palindromic repeats (CRISPR)/CRISPR-associated gene (Cas) system was identified in the same location in both the *S. ratti* and *S. hyicus* genomes. The genes for predicted virulence factors, surface and extracellular proteins found in *S. ratti* CCM 9025^T^ genome are shown in Table 3.

**Figure 5:**
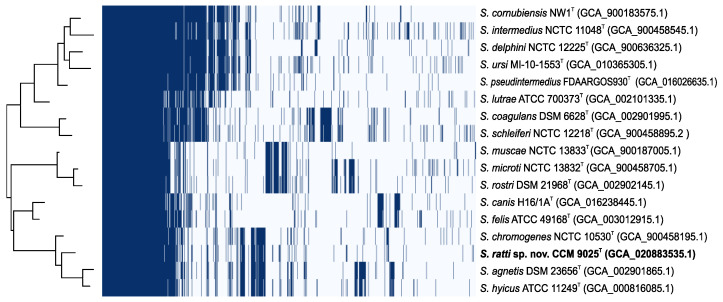
Pangenome analysis of the Hyicus-Intermedius species group showing genomic diversity within the type strains. Gene clusters (*n* = 6056) distinguished at 70% blastp identity were grouped by Roary. The pangenome matrix including 992 core genes present in <16 genomes, 2360 shell genes present in 2–15 genomes and 2704 cloud genes present in <2 genomes where the genes were either present or absent is visualized by the Roary plot on the right. The evolutionary insights between species based on the pangenome is shown by the tree on the left.

The accessory genome is often associated with virulence and antimicrobial resistance and has an important role in the ability of species to colonize particular hosts or persist in the environment. To determine the genomic diversity within all Hyicus-Intermedius species group representatives, the pangenome was analyzed. Type strains within the group have shown an extensive accessory genome whose profile correlates well with the phylogenetic relationship of individual species (Figure 5). The comparison indicates a distinct accessory genes group which is shared with *S. hyicus*, *S. chromogenes* and *S. agnetis* species (Figure 5).

The genome harbors various types of insertion sequences (ISs) from the IS200/IS605, IS3 and IS6 families. In addition to the above ISs, a 9.1-kb composite transposon with 28.3% G+C content is integrated into the genome and flanked by two elements from the IS3 family (Figure 4). The transposon encodes a gene for the radical S-adenosyl-L-methionine (SAM) enzyme (LN051_08115), which is involved in a number of metabolic processes, including post-transcriptional and post-translational modifications, and a gene encoding a YcaO–like protein (LN051_08100) which is responsible for the synthesis of thiazole/oxazole-modified microcin antibiotics [39]. The SagB/ThcOx family dehydrogenase gene (LN051_08090) for a membrane-associated N-acetylglucosaminidase that cleaves polymerized glycan strands to their physiological length and a major facilitator superfamily (MFS) transporter gene (LN051_08095), are also part of the transposon. Since these genes are linked to modulating antibiotic resistance in methicillin-resistant *S. aureus* [40], we hypothesize that these genes may be responsible for the penicillin resistance of this strain.

One prophage designated vB_SraS_LR1 with a typical siphoviral modular structure is integrated at the 13-bp-long putative *att* site 5′ AAAATCAACYTTT 3′ adjacent to the tRNA^Arg^ gene (locus tag LN051_09185) and exhibits 75.9% identity and 43% coverage with the *S. hyicus* phage EW (= RG = NCTC 9856) [41], which was previously misclassified according to its genomic sequence [42] as an *S. aureus* bacteriophage belonging to the *Phietavirus* genus [43]. Moderate similarity was found (77.3% identity and 30% coverage) to the *S. hyicus* phage PMBT9 from the *Siphoviridae* family [44].

**Figure 6:**
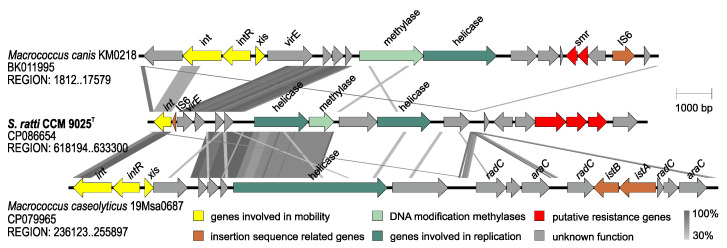
Structure comparison of the phage-inducible chromosomal island SrRI_CCM9025_ from *Staphylococcus ratti* CCM 9025^T^, the partial sequence of McRI_msr_-like island from *Macrococcus canis* KM0218 and the homologous genomic region from *Macrococcus caseolyticus* 19Msa0687. GenBank accession numbers and depicted regions are indicated below the species designation. The Blast map shows protein identities above 30%. The genes are color coded by their function as in the legend.

A 15-kb long phage-inducible chromosomal island designated SrRI_CCM9025_ was identified in the genome of CCM 9025^T^ (Figure 4). It has 30.1% G+C content and harbours a site-specific integrase (LN051_02785), IS6 family transposase (LN051_02790), the gene *virE* encoding virulence-associated E family protein (LN051_02795), and new putative phosphotransferase genes which may be related to antimicrobial resistance. SrRI_CCM9025_ exhibits partial sequence similarity to the *Macrococcus* island McRI*_msr_* [45] in the proteins for integration and replication but otherwise demonstrates a distinct gene structure (Figure 6). It is integrated adjacent to the *lacA*, *lacB*, *lacD*, *lacG* genes and genes for lactose/cellobiose-specific phosphotransferase system genes, which are required for galactose 6-phosphate isomerase activity, described as part of the *S. aureus* lactose operon *lacABCDFEG* [46].

Despite the fact that mobile elements were found in the genome, the identification of the CRISPR/Cas system in the *S. ratti* CCM 9025^T^ genome is consistent with the need to limit the uptake of foreign DNA. Strain CCM 9025^T^ carries a 431-bp-long CRISPR loci with 6 spacers flanked by 36-bp-long direct repeats (DR: GTTTTAGTACTCTGTAATTTTAGTATAAGGTATTC) and putative *cas* genes encoding CRISPR-associated endonuclease Cas9 and the proteins Cas1, Cas2 and Csn1 typical for CRISPR system type II-A. Cas9 exhibited 79% amino acid identity to the type II CRISPR-associated Cas9 of *S. agnetis* (WP_107390356) [47] and *S. chromogenes* (WP_145399953), and 74% amino acid identity to Cas9 of *S. hyicus* ATCC 11249^T^ (WP_167696241) [48]. Spacers target bacteriophage-related sequences, but no significant similarity to staphylococcal phage genomes was found, indicating a gap in the knowledge of phages infecting this host.

### 2.4. Description of Staphylococcus ratti sp. nov.

*Staphylococcus ratti* (rat’ti. L. gen. n. *ratti* of the rat) cells are Gram-stain-positive spherical cocci occurring predominantly in pairs and clusters. They are non-spore-forming and non-motile. Colonies on tryptic soy agar (TSA) are circular with a whole margin and are flat, smooth, shiny, 1–2 mm in diameter, aerobic and white. They demonstrate positive hemolytic activity on blood agar. Growth occurs in the presence of 15% NaCl at 20 °C and 45 °C, but not at 15 °C or 48 °C. Cells contain catalase, arginine dihydrolase, urease, β-glucuronidase, and hyaluronidase, and demonstrate nitrate reduction and hydrolysis of gelatin and DNA. Hydrolysis of esculin and Tween 80 is negative. Cells demonstrate weak anaerobic growth in thioglycolate medium and are coagulase-, thermostable nuclease-, oxidase-, pyrrolidonyl arylamidase-, Voges–Proskauer test (acetoin)- and ornithine decarboxylase-negative. Cells are susceptible to furazolidone (100 µg) and resistant to bacitracin (10 IU). Cells are acid phosphatase-, alkaline phosphatase-, esterase (C4)-, esterase lipase (C8)- and α-chymotrypsin (weak)-positive in the API ZYM kit. Cells are lipase (C14)-, leucine arylamidase-, valine arylamidase-, cystine arylamidase-, trypsin-, naphthol-AS-Bi-phosphohydrolase-, α-galactosidase-, β-galactosidase-, α-glucosidase-, β-glucosidase-, N-acetyl-β-glucosaminidase-, α-mannosidase- and α-fucosidase-negative with the API ZYM kit. Acid is produced from glycerol, ribose, galactose (weak), D-glucose, D-fructose, mannose, N-acetyl glucosamine, lactose, sucrose, and trehalose. Acid is not produced from erythritol, D-arabinose, L-arabinose, D-xylose, L-xylose, adonitol, β-methyl-D-xyloside, sorbose, rhamnose, dulcitol, inositol, mannitol, sorbitol, α-methyl-D-mannoside, α-methyl-D-glucoside, amygdaline, arbutine, salicin, cellobiose, maltose, melibiose, inulin, melezitose, D-raffinose, starch, glycogen, xylitol, β-gentiobiose, D-turanose, D-lyxose, D-tagatose, D-fucose, L-fucose, D-arabitol, L-arabitol, gluconate, 2 keto-gluconate, and 5 keto-gluconate. Cells are susceptible to ceftazidin, cephalothin, erythromycin, gentamicin, chloramphenicol, imipenem, kanamycin, neomycin, novobiocin, oxacillin, trimethoprim, sulphamethoxazole/trimethoprim (cotrimoxazole), tetracycline rifampicin, vancomycin and fusidic acid. Cells are resistant to ampicillin, clindamycin, penicillin G, and polymyxin B. Ciprofloxacin resistance is intermediate. Cells are susceptible to lysostaphin but resistant to lysozyme.

Utilisation of D-trehalose, sucrose, β-methyl-D-glucoside, N-acetyl-D-glucosamine, N-acetyl neuraminic acid, α-D-glucose, D-mannose, D-fructose, glycerol, D-glucose-6-PO_4_, L-histidine, L-serine, pectin and acetic acid is positive, and utilisation of D-turanose is borderline with Biolog MicroPlate GEN III, protocol A. There is negative utilisation of dextrin, D-maltose, D-cellobiose, gentiobiose, stachyose, D-raffinose, α-D-lactose, D-melibiose, D-salicin, N-acetyl-β-D-mannosamine, N-acetyl-D-galactosamine, D-galactose, 3-methyl glucose, D-fucose, L-fucose, L-rhamnose, inosine, D-sorbitol, D-mannitol, D-arabitol, myo-inositol, D-fructose-6-PO_4_, D-aspartic acid, D-serine, gelatin, glycyl-L-proline, L-alanine, L-arginine, L-aspartic acid, L-glutamic acid, L-pyroglutamic acid, D-galacturonic acid, D-galactonic acid lactone, D-gluconic acid, D-glucuronic acid, glucuronamide, mucic acid, quinic acid, D-saccharic acid, p-hydroxy phenylacetic acid, methyl pyruvate, D-lactic acid methyl ester, L-lactic acid, citric acid, α-keto glutaric acid, D-malic acid, L-malic acid, bromo-succinic acid, tween 40, γ-amino-butyric acid, α-hydroxy-butyric acid, β-hydroxy-D,L-butyric acid, α-keto butyric acid, acetoacetic acid, propionic acid and formic acid.

The peptidoglycan is of the type A3α L-Lys–Gly_3–4_, MK-7 is the major menaquinone, and the major fatty acids are iso-C_15:0_, anteiso-C_15:0_, iso-C_17:0_ and anteiso-C_17:0_.

The type strain is NRL/St 03/464^T^ (= CCM 9025^T^ = LMG 31873^T^ = DSM 111348^T^), isolated in 2003 from a swab from the nostrils of a healthy laboratory rat. The DNA G+C content of the type strain is 36.1 mol%, calculated from the whole genomic sequence. The GenBank/ENA/DDBJ accession number for the 16S rRNA gene is OL352091. The complete genome sequence is available under GenBank accession number CP086654.

## 3. Materials and Methods

### 3.1. Bacterial Strains and Cultivation

The isolate NRL St 03/464^T^ was isolated from a single animal and maintained as glycerol stock at −70 °C until analyzed. Reference strains of the phylogenetic relatives *S. agnetis* CCM 8869^T^, *S. hyicus* CCM 2368^T^, and *S. chromogenes* CCM 3387^T^ were obtained from the Czech Collection of Microorganisms (Brno, Czech Republic). All cultivations were performed at 30 °C for 24 h unless stated otherwise in the test specifications. Type strain *Staphylococcus ratti* NRL/St 03/464^T^ has been deposited as publicly accessible in the Czech Collection of Microorganisms (CCM), the German Collection of Microorganisms and Cell Cultures (DSMZ), and the BCCM/LMG Bacteria collection.

### 3.2. Phenotypic Characterization

Extensive phenotypic characterization using the commercial kits STAPHYtest 24 (Erba Lachema, Brno, Czechia) and API ZYM (bioMérieux, Marcy l’Etoile, France), phenotypic fingerprinting using the Biolog system with the identification test panel GEN III MicroPlate (Biolog, Hayward, CA, USA), and conventional biochemical, physiological, and growth tests relevant for the genus *Staphylococcus* were done as described previously [49,50,51]. The antibiotic resistance pattern was tested by the disc diffusion method on Mueller–Hinton agar (Oxoid, Basingstoke, UK). A set of discs (Oxoid) generally used for Gram-positive cocci were applied: ampicillin (10 µg), oxacillin (1 µg), ceftazidime (30 µg), cephalothin (30 µg), ciprofloxacin (5 µg), clindamycin (2 µg), erythromycin (15 µg), gentamicin (10 µg), chloramphenicol (30 µg), imipenem (10 µg), kanamycin (30 µg), neomycin (10 µg), novobiocin (5 µg), penicillin G (1 IU), rifampicin (5 µg), trimethoprim (5 µg), cotrimoxazole (25 µg), tetracycline (30 µg), vancomycin (30 µg), fusidic acid (10 µg) and polymyxin B (300 U). EUCAST standards and manufacturer’s recommendations (Oxoid) were strictly followed for cultivation and inhibition zone diameter measurement [52].

### 3.3. Transmission Electron Microscopy

A 200-mesh carbon/formvar-coated grid was placed on a drop of suspension of bacteria in water for 20 min. Bacterial cells located on the grid were negatively stained with 2% ammonium molybdate and treated with UV light. A Morgagni 268D Philips (ThermoFisher Scientific, Amsterdam, The Netherlands) transmission electron microscope was used to visualize bacterial cells.

### 3.4. Matrix-Assisted Laser Desorption/Ionization Time-of-Flight Mass Spectrometry (MALDI-TOF MS) Analysis

MALDI-TOF MS was performed in automatic acquisition mode as described previously [53] in a Microflex LT MALDI-TOF spectrometer (Bruker Daltonics, Bremen, Germany) by using MBT Compass 4.1 software (Bruker Daltonics). Identification was made using the Bruker’s database MBT Compass Library Revision L 2020 (9607 MSP).

### 3.5. Chemotaxonomic Analyses

Cellular fatty acids (FAs) were extracted from all compared *Staphylococcus* strains grown on the Trypticase soy broth agar (TSBA) plates under the same cultivation conditions with a cultivation temperature of 37 °C for 24 h to reach the late-exponential stage of growth according to the four-quadrant streak method. The extraction of cellular FAs was performed according to the standard protocol recommended by the MIDI Microbial Identification System [54]. Extracted FAs were identified using an Agilent 7890B gas chromatograph (Agilent Technologies, Santa Clara, CA, USA) according to the Standard Protocol of the Sherlock Identification System (MIDI Sherlock version 6.2, MIDI database RTSBA version 6.21).

Isolation and structure analyses of the peptidoglycan were performed according to published protocols and some modifications [35,55,56]. In brief, the amino acid composition of total hydrolysate (4 N HCl at 100 °C for 16 h) of the peptidoglycan was analyzed by gas chromatography/mass spectrometry (protocol 10 [35]). The partial hydrolysate (4 N HCl, 100 °C, 45 min) of the peptidoglycan was analyzed by high-resolution liquid chromatography mass spectrometry (LC-MS) as described in [35,55]. Enantiomeric analysis was performed by liquid chromatography as described recently [56].

Respiratory quinones were extracted and analyzed as described previously [57]. Their identity was confirmed by mass spectrometry as described previously [55].

### 3.6. Genotypic Analysis by (GTG)_5_-PCR Fingerprinting

Rep-PCR fingerprinting using the (GTG)_5_ primer was carried out according to Švec et al. [58]. Numerical analysis of the (GTG)_5_-PCR fingerprints was done using BioNumerics version 7.6 (Applied Maths, Kortrijk, Belgium).

### 3.7. Phylogenetic Analysis Based on 16S rRNA, Housekeeping Genes and Core Genome

The 16S rRNA gene sequences were amplified from crude boiled cell extracts and sequenced by Sanger sequencing in the Eurofins MWG Operon sequencing facility (Ebersberg, Germany) with previously described primers [59]. The partial *rpoB* gene was sequenced as described previously [60]. Initial identification of the strain to the genus level was based on pairwise sequence alignment and calculation of similarity values with the algorithm used in the EzBioCloud database v.2021.07.07 [61]. 16S rRNA gene sequences obtained from PCR products were aligned with those extracted from WGS data using RNAmmer version 1.2 [62]. The multilocus sequence data of six housekeeping genes that are commonly used in phylogenetic studies of the *Staphylococcus* genus were extracted from whole-genome sequence assemblies of type strains available in the NCBI Assembly resource [63], including the NCTC 3000 project (https://www.sanger.ac.uk/resources/downloads/bacteria/nctc/ accessed on 27 October 2021) and FDA-ARGOS project [64]. The partial gene sequences used correspond to the following gene coordinates of *S. aureus*: 1420..1974 for *rpoB*, 270..826 for *groEL*, 23..911 for *dnaJ*, 49..929 for *gap*, 383..1032 for *tufA*, and 50..480 for the *sodA* gene. The phylogenetic analyses were performed with the software MEGA X [65]. Genetic distances were corrected using the Tamura–Nei model [66], and the evolutionary history was inferred using the maximum likelihood (ML) and neighbor-joining (NJ) methods using a bootstrap test based on 500 replications [67]. The up-to-date bacterial core gene (UBCG) pipeline version 3.0 was used for whole-genome phylogenetic analysis based on core gene sequences [68].

The ANI and dDDH values were calculated using the web-based genome-to-genome distance calculator (GGDC) version 3.0 [69] and FastANI [70], respectively.

### 3.8. Genome Sequencing and Bioinformatics Analyses

Total genomic DNA was extracted using a GenElute Bacterial Genomic DNA kit (Sigma-Aldrich, St. Louis, MO, USA) from pure culture colonies cultivated on Colombia sheep blood agar (Oxoid). The preparation of DNA libraries with a Nextera XT DNA Library Preparation Kit (Illumina, San Diego, CA, USA) and whole-genome sequencing on the Illumina platform were conducted externally (LGC Genomics, Berlin, Germany) using 2 × 150 bp paired-end reads on the NextSeq sequencing platform (Illumina).

For sequencing using the Oxford Nanopore platform, bacterial DNA was isolated as described previously [71]. The library was prepared using the SQK-RBK004 rapid barcoding kit (Oxford Nanopore Technologies, Oxford, UK) according to the manufacturer’s instructions. Libraries were sequenced with FLO-MIN106 flow cells (R9.4.1) in a MinION device (Oxford Nanopore Technologies, Oxford, UK). The device was controlled with the software MinKNOW version 4.1.2 (Oxford Nanopore Technologies, Oxford, UK).

Basecalling, demultiplexing and barcode trimming were performed using stand-alone ONT Guppy software version 5.0.11 using the config file dna_r9.4.1_450bps_sup.cfg with the default minimum q-score threshold, i.e., 10. The MinION reads were subsequently filtered by mapping to Illumina reads using Filtlong version 0.2.1 (https://github.com/rrwick/Filtlong accessed on 20 September 2021) with a minimum length of 1500 bp and quality threshold set to 95%. Only data that exceeded these thresholds was used in the assembly. The quality of reads was assessed with FastQC version 0.11.9 (http://www.bioinformatics.babraham.ac.uk/projects/fastqc accessed on 20 September 2021) and NanoStat [72]. Complete bacterial genome sequences were obtained using a hybrid assembly with Unicycler version 0.4.9 [73] using SPAdes version 3.12.0 [74], and the parameters chosen were bold mode and k-mers 21,55,77,99,127. The resulting assembly was polished with Pilon version 1.24 [75].

For pangenome analysis, the complete genomes were initially annotated with Prokka version 1.14.6 [76] and clustered with Roary [77]; the results were then visualized with the script roary_plots.py, which is provided in the Roary package. Further, the genome was annotated using the NCBI Prokaryotic Genome Annotation Pipeline [78]. Sequences were manipulated and inspected in the cross-platform bioinformatics software Ugene version 38.1 [79]. The multiple sequence alignment was visualised using EasyFig version 2.2.5 [80]. Gene content was further examined manually with NCBI BLAST (https://blast.ncbi.nlm.nih.gov accessed on 4 November 2021), and VGEs were identified with PHASTER [81], PhiSpy version 3.4 [82], IslandViewer 4 [83], and ISFinder [84]. The CRISPR/Cas system was characterized with CRISPRCasTyper [85]. Virulence factors were predicted using the VFanalyzer tool available at the Virulence Factors Database [86].

## Figures and Tables

**Table 1:** Phenotypic differentiation of *Staphylococcus ratti* sp. nov. from closely related *Staphylococcus* spp. type strains.

Feature	*S. ratti* sp. nov. CCM 9025^T^	*S. agnetis* CCM 8869^T^	*S. hyicus* CCM 2368^T^	*S. chromogenes* CCM 3387^T^
Pigment	white	white	white	orange
Growth at 15 °C	−	−	+	+
Growth in 15% NaCl	+	+	+	−
Hemolysis	+	−	−	−
Voges–Proskauer test ^1^	−	+	−	−
Acid from trehalose	+	−	+	+
Tween 80 hydrolysis	−	+	+	−
Alkaline phosphatase	+	−	+	+
β-glucuronidase ^2^	+	w	+	−
Urease	+	−	−	+

+, positive; w, weakly positive; −, negative; all data were taken from this study. ^1^ Tube test; ^2^ STAPHYtest 24 kit.

**Table 2:** Cellular fatty acid composition (as a percentage of the total) of *S. ratti* sp. nov. CCM 9025^T^*, S. chromogenes* CCM 3387^T^, *S. agnetis* CCM 8869^T^ and *S. hyicus* CCM 2368^T^. Values of less than 1% are not shown.

Fatty Acid	*S. ratti* sp. nov. CCM 9025^T^	*S. chromogenes* CCM 3387^T^	*S. agnetis* CCM 8869^T^	*S. hyicus* CCM 2368^T^
iso-C_14:0_	TR	1.0	TR	TR
C_14:0_	TR	TR	1.0	1.0
iso-C_15:0_	35.0	16.5	43.9	33.7
anteiso-C_15:0_	24.1	36.4	25.1	32.5
iso-C_16:0_	1.5	1.5	TR	1.2
C_16:0_	1.5	1.5	2.4	2.7
iso-C_17:0_	10.8	6.0	9.2	7.4
anteiso-C_17:0_	10.2	13.4	4.8	6.9
C_18:0_	2.6	5.5	2.9	4.0
iso-C_19:0_	4.4	3.2	2.1	2.1
anteiso-C_19:0_	2.3	2.9	TR	1.0
C_20:0_	5.8	10.4	6.1	6.2

TR, trace amounts < 1%. Cultivation on TSBA medium for 24 h at 37 °C.

**Table 3:** Candidate virulence factors predicted in *Staphylococcus ratti* sp. nov. Locus tags in the genome representing homologs with known and previously predicted virulence factors are shown.

Function and Role	Virulence Factors	Related Genes	Prediction in CCM 9025^T^ Genome
Adherence	Clumping factor B	*clfB*	LN051_01230
	Fibronectin binding proteins	*fnbA*	LN051_04265
	Ser-Asp rich fibrinogen-binding proteins	*sdrC*	LN051_00305
Enzymes	Cysteine protease	*sspB*	LN051_01195
	Hyaluronate lyase	*hysA*	LN051_02175
	Lipase	*geh*	LN051_01425
	Thermonuclease	*nuc*	LN051_06665
Secretion system	Type VII secretion system	*esaA*	LN051_10885; LN051_10890
		*esaB*	LN051_10875
		*essC*	LN051_10865
		*esxA*	LN051_10895
Surface protein anchoring	Lipoprotein diacylglyceryl transferase	*lgt*	LN051_09220
	Lipoprotein-specific signal peptidase II	*lspA*	LN051_07315
Immune evasion	Capsule	Undetermined	LN051_02355; LN051_03230; LN051_07970
Toxin	β-hemolysin	*hlb*	LN051_01075

## Data Availability

The complete genome sequence of strain NRL/St 03/464^T^ (= CCM 9025^T^) has been deposited in GenBank/ENA/DDBJ under accession number CP086654 (chromosome) and CP086655 and CP086656 (plasmids). The accession number for the 16S rRNA gene is OL352091. The associated BioProject and BioSample accession numbers are PRJNA779216 and SAMN22456156, respectively. The raw reads were deposited into the SRA database with accession numbers SRR16917662 (Illumina NextSeq 500) and SRR16917661 (Oxford Nanopore MinION).
